# The Toxicity of Protein Aggregates: New Insights into the Mechanisms

**DOI:** 10.3390/ijms24097974

**Published:** 2023-04-28

**Authors:** Alessandra Bigi, Eva Lombardo, Roberta Cascella, Cristina Cecchi

**Affiliations:** Department of Experimental and Clinical Biomedical Sciences, Section of Biochemistry, University of Florence, 50134 Florence, Italy; alessandra.bigi@unifi.it (A.B.); eva.lombardo@unifi.it (E.L.)

**Figure 1:**
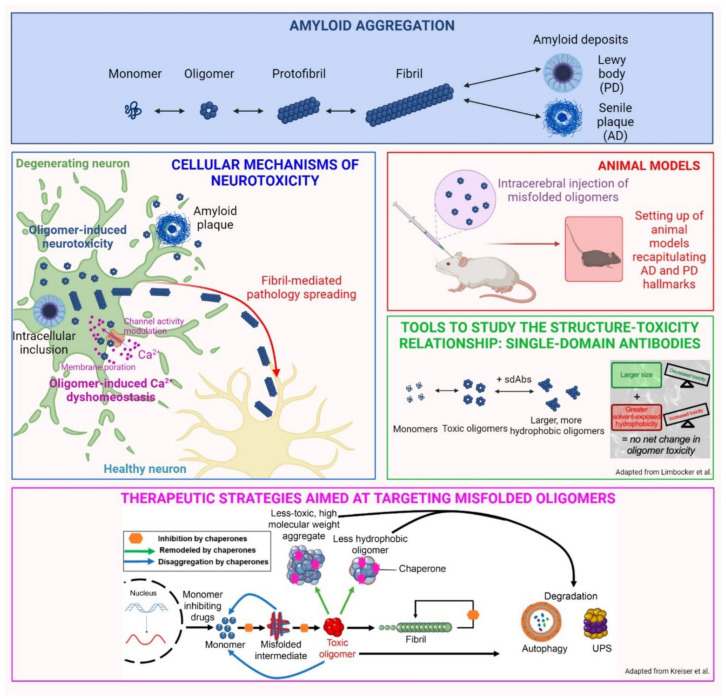
Overview of the main topics developed in the Special Issue. Adapted from Limbocker et al. [9] and Kreiser et al. [11].

The aberrant aggregation of specific peptides and proteins is the common feature of a range of more than 50 human pathologies, collectively referred to as protein misfolding diseases. Among them, neurodegenerative disorders such as Alzheimer’s and Parkinson’s diseases (AD and PD, respectively) represent a real burden in terms of disability and health care cost [1]. Specifically, AD is characterized by the accumulation of aberrant aggregates of the amyloid-β peptide (Aβ) and hyperphosphorylated tau protein, whereas PD is defined by the aggregation and accumulation of α-synuclein (αS). In these conditions, misfolded monomeric proteins undergo conformational shifts, facilitating the aggregation and formation of insoluble amyloid fibrils (Figure 1, “amyloid aggregation” panel). Although the presence of inclusions is considered to be a cardinal pathological hallmark, their abundance does not directly correlate with the disease phenotype observed in patients [1]. Accordingly, a robust body of evidence indicates that oligomeric species formed at the early stages of the aggregation process are primarily responsible for neuronal toxicity [2]. Many hypotheses have been proposed to explain the molecular pathogenesis of neurodegeneration, but despite considerable efforts by researchers in recent decades, it has not been completely elucidated and further investigations are still necessary.

The Special Issue entitled “Protein Aggregates Toxicity: New Insights into the Mechanisms” published in *International Journal of Molecular Sciences* includes different contributions: two original articles and three reviews providing new information about the molecular mechanisms of neurotoxicity caused by misfolded proteins in neurodegenerative disorders, as well as possible therapeutic strategies to counteract their detrimental effects.

A summary of the current state of knowledge on αS aggregates has been issued by Gracia et al. in the review entitled “Multiplicity of α-synuclein aggregated species and their possible roles in disease” [3], in which they recapitulated the different processes that can take place during αS aggregation, including primary and secondary nucleation, ultimately giving rise to multiple amyloid polymorphs and intermediate species. They emphasized the proposition that oligomeric species have more inherent abilities to promptly induce cellular dysfunction and neurotoxicity, while fibrillar conformers are more effective in spreading from neuron to neuron, thus propagating neurodegeneration, as depicted in Figure 1, “cellular mechanisms of neurotoxicity”. These conclusions arise from experimental evidences by several researchers, including ourselves, on the prominent neurotoxic potential of oligomeric species, which are not only formed during αS aggregation, but are also released by mature fibrils upon their interaction with the neuronal membrane [4,5,6,7]. The review by Gracia et al. presents the state-of-the-art knowledge on αS aggregation and toxicity, which is extremely useful for hypothesizing therapeutic drugs and diagnostic tools for accurate diagnoses. L. Boi and coworkers, in the original research article entitled “Modeling Parkinson’s disease neuropathology and symptoms by intranigral inoculation of preformed human α-synuclein oligomers” [8], tested soluble αS oligomers in vivo to assess their neurotoxic potential, as well as their ability to induce a pathological scenario that recapitulates PD features. Their inoculation resulted in the extensive phosphorylation of Ser-129 of αS, which is a cardinal pathological hallmark of PD, as well as in an intense neuroinflammatory response, accompanied by gradual nigrostriatal degeneration, finally leading to motor and cognitive impairment. The study provides a reproducible model of PD, which recapitulates the hallmarks of the disease and will potentially be useful as a tool for testing new therapies (Figure 1, “animal models”). Moreover, the neurotoxic potential of αS oligomers was confirmed again, pointing out the urgency of identifying possible therapeutic approaches selectively targeting this type of misfolded conformer.

The neurotoxicity of oligomeric aggregates is not an exclusive feature of PD, but it has also been described for Aβ peptide in AD and corroborated by many papers, as described in Bigi et al., 2022 [2]. Though their role in pathology has been defined, the isolation and characterization of species from the body fluids of patients remain important challenges to overcome, given their low concentration and transient and heterogeneous nature. R. Limbocker and coworkers, in the original article entitled “Rationally designed antibodies as research tools to study the structure–toxicity relationship of amyloid-β oligomers” [9], proposed the use of rationally designed single-domain antibodies (DesAbs) to define the relationship between the structure and toxicity of Zn^2+^-stabilized Aβ_40_ oligomers. Specifically, they revealed that DesAb_18–24_ and DesAb_34–40_ (raised against residues 18–24 and 34–40 of Aβ_40_, respectively) increase the size and the exposed hydrophobicity of oligomers, which are widely recognized key determinants of neurotoxicity. Accordingly, these increments neutralize each other in terms of neurotoxic effects: while elevations of hydrophobicity are associated with increased cytotoxicity, the size increase is linked to reduced neurotoxicity. The authors propose the use of DesAbs as molecular tools to target with high specificity the disease-related proteins to define a more convincing structure–toxicity relationship. The small size and the high solvent-exposed hydrophobicity are key features of oligomeric conformers, enabling their aberrant interaction with neuronal membranes, in which they can induce changes in permeability and fluidity or interact with specific exposed receptors, aberrantly modulating their activity (Figure 1, “tools to study the structure-toxicity relationship”). One of the prominent consequences of these interactions is the alteration of Ca^2+^ homeostasis, an early event in the cascade of neuronal alterations, underlying the cytotoxicity induced by misfolded Aβ aggregates and hyperphosphorylated tau. This was the object of a review entitled “Calcium dyshomeostasis in Alzheimer’s disease pathogenesis” by Cascella and Cecchi [10]. They described the ability of misfolded Aβ to induce the hyperactivation of membrane calcium channels, such as the N-methyl D-aspartate receptor (NMDAR), the α -amino-3- hydroxy-5-methyl-4-isoxazolepropionic acid receptor (AMPAR), as well as voltage-gated channels. At the same time, these species can cause Ca^2+^ overload, forming cation-selective pores into the plasma membrane, as depicted in Figure 1, “cellular mechanisms of neurotoxicity”. Their capability to induce the excessive release of Ca^2+^ from intracellular stores, such as the endoplasmic reticulum and mitochondria, has been described as an early causative event in AD. Further insights into the molecular mechanisms of calcium perturbation are required for the development of possible therapeutic strategies; not surprisingly, one of the few drugs approved by regulatory agencies is memantine, which targets NMDARs to reduce calcium dyshomeostasis in AD. At the same time, several other strategies to suppress oligomer toxicity were highlighted in the review by R. P. Kreiser, entitled “Therapeutic strategies to reduce the toxicity of misfolded protein oligomers” [11]. In particular, the authors reviewed several approaches and related compounds for their ability to modulate the kinetics of oligomer assembly (via inhibiting, enhancing, or redirecting the aggregation process), to modulate their biophysical properties (such as size and hydrophobicity), to protect cell membranes by displacing oligomeric species, and to enhance the cellular proteostasis system. Different therapeutic strategies were analyzed and compared to develop compounds to combat the toxic effects of oligomers in neurodegenerative diseases, as represented in Figure 1, lower panel. For all the described strategies, the specificity for oligomeric aggregates remains a critical challenge in the design of effective therapeutics. Among the possible therapeutic agents, naturally occurring aminosterols, such as squalamine or trodusquemine, were recently reported to modulate the aggregation of Aβ and αS, thereby attenuating the rate of the oligomerization reaction. These molecules were also described for their ability to inhibit the binding of toxic oligomers to the plasma membrane of neuronal cells [12].

Overall, this Special Issue collects advanced knowledge about specific mechanisms underlying the pathogenesis and progression of protein misfolding diseases linked to neurodegeneration, as well as the current and promising diagnostic and therapeutic approaches.
